# Supplementary material for: Galectin-9 as a Potential Modulator of Lymphocyte Adhesion to Endothelium via Binding to Blood Group H Glycan
Source: Biomolecules. 2023 Jul 26;13(8):1166. doi: 10.3390/biom13081166 (PMC10452646; doi:10.3390/biom13081166)
Supplement: Supplementary file 1 [file biomolecules-13-01166-s001.zip › biomolecules-2468335-supplementary.pdf]

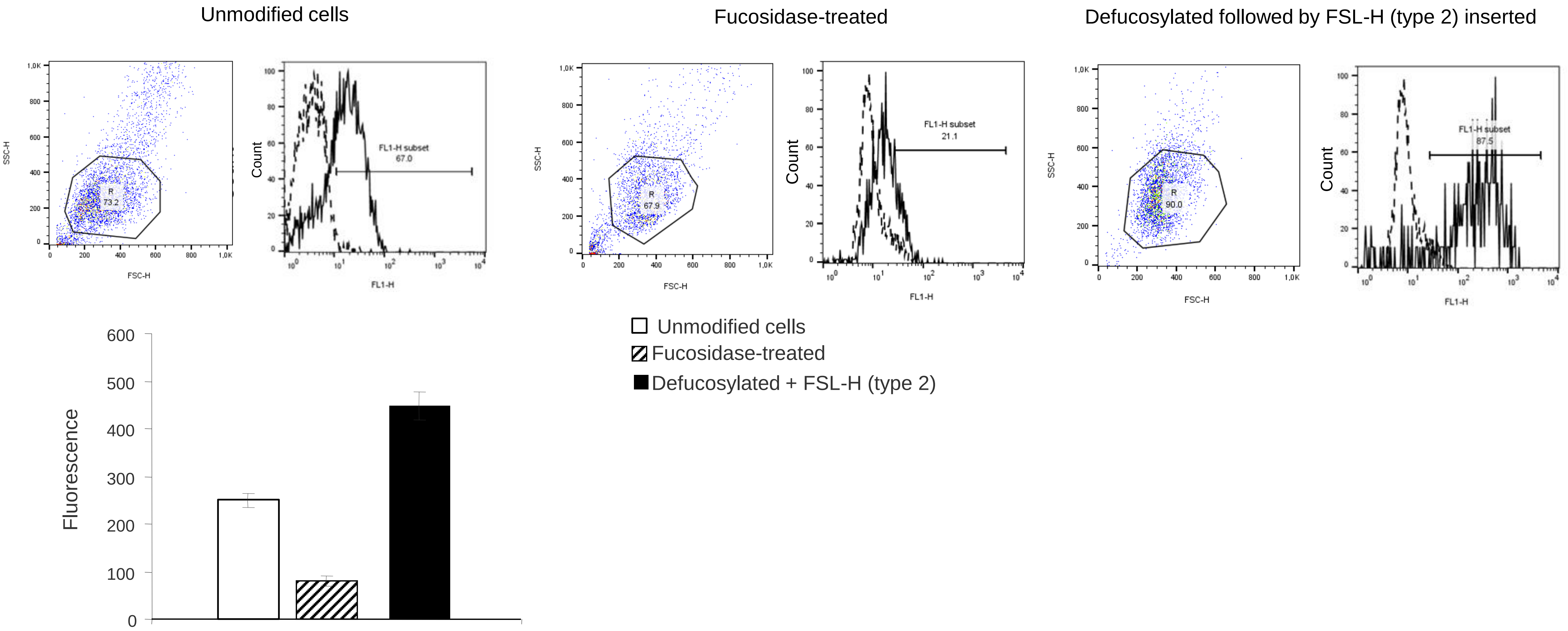

Figure S1. Efficacy of fucosidase treatment and insertion of FSL-H (type 2) into defucosylated EAhy 926 cells as controlled by UEA I binding using flow cytometry. Results shown include the upper panel: dot plots (SSC-H vs. FSC-H), fluorograms (the log of fluorescence intensity FL-1, X-axis were plotted against cell number Y-axis), the number given for the black curve represents the percentage of cells reactive with the UEA-I, bottom panel graph (Y-axis represents calculated fluorescence as described in Material and Methods), data from three experiments, error bars represent the standard deviation

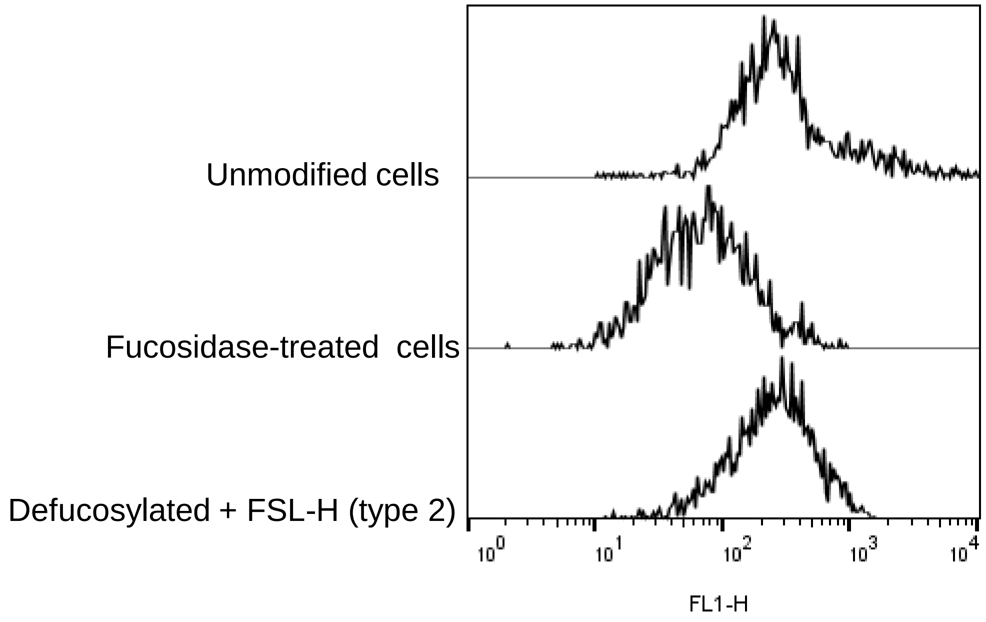

Figure S2. Binding of gal-9 to unmodified, fucosidase-treated, and after FSL-H (type 2) insertion, flow cytometry analysis. Analysis of inserted FSL-H (type 2) was performed as described in Materials and Methods; the log of fluorescence intensity (FL-1, X-axis) was plotted against cell number (Y-axis).
